# The Relationship Between Children’s Indoor Loose Parts Play and Cognitive Development: A Systematic Review

**DOI:** 10.3390/jintelligence13050052

**Published:** 2025-04-23

**Authors:** Ozlem Cankaya, Mackenzie Martin, Dana Haugen

**Affiliations:** 1 Early Childhood Curriculum Studies, MacEwan University, Edmonton, AB T5J 4S2, Canada; 2 Community University Partnership, University of Alberta, Edmonton, AB T6G 1C9, Canada; mackenzie.martin@ualberta.ca; 3 Department of Educational Counselling, University of Lethbridge, Lethbridge, AB T1K 3M4, Canada; dana.haugen@uleth.ca

**Keywords:** play, cognitive development, loose parts, loose parts play, LPP, indoor play

## Abstract

Children’s engagement with toys and play materials can contribute to the foundational cognitive processes that drive learning. Loose parts are interactive, open-ended materials originally not designed as toys but can be incorporated into children’s play (e.g., acorns, cardboard, and fabric). Practitioners and researchers widely endorse loose parts for fostering creativity, divergent thinking, and problem-solving skills. Despite these recommendations, research on their specific role in young children’s cognitive development remains limited. This systematic review examines how indoor loose parts play has been studied in relation to young children’s (0–6 years) cognitive development. Following PRISMA guidelines, searches in bibliographic databases and forward and backward citation tracking identified 5721 studies published until December 2024. We identified 25 studies and evaluated the quality and risk of bias. Studies focused on children’s general cognitive outcomes, language development, and specific cognitive subdomains, with many reporting positive associations between children’s play materials and cognitive development. However, five studies found no such associations, and another seven did not address the relationship between play materials and outcomes. Despite methodological variation across studies, our systematic review identified a relationship between play materials similar to loose parts and children’s problem-solving, creativity, academic skills (reading and math), and both convergent and divergent thinking. Notably, only one study explicitly used the term “loose parts.”Our review identified empirical and methodological gaps regarding the relationship between play materials and cognitive development, which can inform future research.

## 1. Introduction

Children’s play can involve various behaviours and activities, resulting in varied developmental and learning outcomes. These developmental outcomes depend on many factors, such as the type of toys and play materials used, a child’s age, social interactions, and the quality of the learning environment (Lin and Li 2019; Howe et al. 2022; Rubin 2001; Wood 2013). The properties of toys and play materials in the environment can impact children’s play behaviours, engagement, and duration. There are a growing number of calls to enrich young children’s indoor play, explorations, and learning using loose parts (Beaudin 2021; Beloglovsky and Daly 2015, 2016; Caldwell 2016; Casey and Robertson 2019; Daly and Beloglovsky 2014; Rawstrone 2020). Loose parts are interactive, natural, and manufactured materials that can be manipulated with limitless possibilities (Houser et al. 2019). Children’s loose parts play (LPP) is unique, involving various toys and materials that can be used in combination or isolation (e.g., cardboard, sticks, pipes, sand, and beads) but are generally not intended for play (Gull et al. 2020; Houser et al. 2016; Nicholson 1971).

When children engage in play that includes toys, materials, and objects—particularly those that naturally complement each other or can be effectively combined—they are immersed in a learning environment that promotes cognitive development (Cutter-Mackenzie and Edwards 2013; Trawick-Smith 1990; Trawick-Smith et al. 2015). Such play supports essential cognitive processes, including impulse control, behaviour regulation, exploration, problem-solving, attention to outcomes, and social interaction (Park 2019), all of which are foundational to learning (Wolfgang et al. 2001).

Play also serves as a powerful source of intrinsic motivation (Haber et al. 2018; Kidd and Hayden 2015; Malone 1981), vital for long-term academic and personal success (Hewes 2006). Unlike structured learning, which often depends on external rewards, play enables children to engage in learning driven by curiosity and interest (Andersen et al. 2023). This intrinsic motivation, developed through early experiences, tends to persist into adulthood (Andersen and Kiverstein 2024; Cassidy 2000; Gottfried et al. 2001, 2011, 2016). Through these experiences, children explore their environment, test hypotheses, and make discoveries—activities that form the basis for scientific thinking (Andersen and Kiverstein 2024).

LPP can be particularly valuable for children under six in early childhood because it encourages open-ended exploration, allowing children to manipulate materials in ways that support their learning needs, cognitive flexibility, problem-solving, and creativity. Unlike predefined toys, loose parts afford children the autonomy to direct their play and foster decision-making and independent thinking. This capacity for self-directed learning makes play a critical tool for supporting young cognitive development in early childhood education and home environments (Smith and Pellegrini 2013; Zosh et al. 2018).

*What is the relationship between children’s play and cognitive development?*

Given play’s imaginative and flexible nature, it is key in fostering cognitive processes such as problem-solving, generating ideas, and identifying alternative possibilities (Cankaya et al. 2023; Harris 2000). Researchers have examined specific types of play or play forms, such as pretend play, constructive play, and block play, and their impact on children’s cognitive development (Lillard et al. 2013; Smith 2017; Wolfgang et al. 2001). The research on these play types has consistently demonstrated that play serves as a powerful medium through which children build the foundational skills for language development, contributing to broader cognitive outcomes. Pretend play, for example, allows children to exercise their cognitive skills by creating and navigating complex scenarios and inventing rules (Cankaya et al. 2023; Lillard et al. 2013). Play’s exploratory and manipulative nature, particularly through object play, supports children’s scientific reasoning and divergent and convergent thinking. Several studies have linked early object and constructive play to improved developmental outcomes (Caldera et al. 1999; Ness and Farenga 2016; Verdine et al. 2019; Wolfgang et al. 2001). Verdine et al. (2019) highlight that toys with geometric shapes enhance spatial language and interactions. Longitudinal research by Wolfgang et al. (2001) found that children who engaged in complex object play in early childhood demonstrated improved mathematical outcomes later in life. As a result, children’s involvement in various types of play is significant in the early years (Pellegrini and Smith 1998). Play is a crucial mechanism through which children explore, learn, and develop essential cognitive skills that can lay the foundation for academic achievement, learning, and problem-solving abilities later in life (Copple and Bredekamp 2006; Lillard et al. 2013; Miller et al. 2022; Savina 2014; Weisberg et al. 2013).

*Do play materials and toys make a difference in cognitive outcomes?*

Researchers have found that certain toys or play materials can lead to unique cognitive outcomes (e.g., play with blocks, LEGO, or sand in isolation; Iivonen et al. 2025; Kiewra and Veselack 2016; Schulz and Bonawitz 2007; Segatti et al. 2003; Shabazian and Soga 2014; Zippert et al. 2019). For instance, while dolls promote language skills, blocks can increase mathematical knowledge (Hashmi et al. 2020; Verdine et al. 2014). Similarly, children’s dramatic or pretend play allows for imaginative scenarios, taking on different roles, and experimenting with social roles and relationships (Harris 2000). Most crucially, children may take a concrete object or an imaginary role and pretend “as-if” (Harris 2000). This type of play encourages children to think symbolically, promoting perspective-taking, empathy, and understanding of social norms and expectations (Vygotsky 1967). Children learn to navigate complex social interactions, negotiate roles, and regulate emotions in imaginary situations. As loose parts offer multiple opportunities for many play types, engaging with these materials may have the potential to significantly impact children’s cognitive development by enhancing various cognitive skills and abilities (Cankaya et al. 2023).

*What is the status of research on children’s indoor LPP and cognitive outcomes?*

Recently, interest in LPP as a means to enrich children’s play has grown (Beaudin 2021; Beloglovsky and Daly 2015, 2016; Branje et al. 2021; Caldwell 2016; Casey and Robertson 2019; Gold et al. 2020; Gull et al. 2024; Rawstrone 2020). The current attention to this play type is highlighted in early learning curriculum frameworks (e.g., Government of Manitoba 2015; Makovichuk et al. 2014; Nova Scotia Department of Education 2018) as well as statements made by play organizations (e.g., Play Scotland 2022), researchers, policymakers, and educators as a means to facilitate cognitive development and learning (Casey and Robertson 2019; Ocal 2021; Gençer and Avci 2017; Gold et al. 2015, 2020; Gold and Elicker 2020; Sear 2016). Despite extensive recommendations, research on indoor LPP and its impact on children’s cognitive development is sparse, and an overview of the research on the value of this emerging type of play is lacking.

Research on LPP is limited in many respects: most evidence is on outdoor LPP and children’s physical and social development without directly investigating the role of LPP in cognitive outcomes (Branje et al. 2021; Engelen et al. 2013; Flannigan and Dietze 2017; Gull et al. 2019; Houser et al. 2016; Maxwell et al. 2008; Olsen and Smith 2017; Pereira et al. 2024; Ridgers et al. 2011; Spencer et al. 2019; Trina 2022; van Rooijen et al. 2023). One systematic review by Gibson et al. (2017) examined the effects of LPP on various child developmental domains. They concluded that there is insufficient high-quality evidence to determine the impact on children’s cognitive, social, and emotional development (e.g., limited evidence linking LPP to non-physical developmental outcomes). They also found that tools used to measure development were broad spectrum (e.g., social, emotional, physical); models lacked confounding variables; and many studies reported no significant effects on social and academic outcomes (e.g., Bundy et al. 2017; Farmer et al. 2017). Gibson and colleagues’ review is recent. However, the search terms for their review only focused on outdoor play, and they did not examine the literature with similar conceptual ideas to loose parts such as “recycled materials, junk or scrapped materials”. In a non-systematic review, Cankaya et al. (2023) examined the relationship between LPP and cognitive development and synthesized the common play types used with loose parts materials. While they identified studies that supported the benefits of playing with various toys and materials, they noted a lack of empirical evidence to substantiate these claims, as none of the studies were explicitly on LPP.

Only one recent study explicitly examined indoor and outdoor LPP in a structured empirical design. Jaruchainiwat et al. (2024) conducted a quasi-experimental pre-test–post-test study with 147 preschoolers. The study measured creative thinking behaviour (exploration, participation/enjoyment, persistence), social behaviour (social play, emotional regulation, communication), and attention (measured in one-minute increments). The results showed that outdoor LPP significantly enhanced creative thinking, social skills, and attention, whereas indoor LPP improved creative and social behaviour but had no measurable impact on attention. Although this study provides valuable insights, its reliance on researcher-developed instruments to measure cognitive and social behaviours raises concerns about validity and generalizability. Additionally, data collection in indoor conditions was conducted by parents, introducing potential observer bias and inconsistency in assessments. Furthermore, Naish et al. (2023) examined parents’ perceptions of take-home loose parts play kits during the COVID-19 pandemic. Parents reported that the kits supported unstructured play at home, reinforcing its role in children’s engagement and development. While this study also highlights the potential of LPP, the small sample size and qualitative nature limit broader conclusions.

In addition to these studies, there are some conceptual reports, qualitative, and action research on indoor LPP (Fikriyati et al. 2023; Gorrie 2021; Gull et al. 2024; Jannah and Puridawaty 2023; Mukhyar et al. 2023; Smith-Gilman 2018; Sukardjo et al. 2023; Zeng and Ng 2024). These provide important insights into the theories underpinning LPP, ideas for including loose parts in children’s play, and summaries of the potential or perceived benefits of indoor LPP. While LPP may offer benefits, its relationship to children’s cognitive development remains unclear through empirical quantitative studies. This highlights the need for further research to document the conditions under which LPP may be most beneficial and synthesize the existing evidence. Moreover, there is a notable gap in empirical evidence regarding specific cognitive outcomes, including intelligence, executive functioning, problem-solving, and divergent thinking (e.g., Beaudin 2021; Rawstrone 2020).

Our systematic review examines the relationship between loose parts and children’s cognitive development, situating our findings within the Science of Learning framework (Darling-Hammond et al. 2020). According to this framework, children’s learning is shaped by dynamic interactions between their environment, experiences, and cognitive processes, emphasizing that development is an experience-dependent process influenced by social and contextual factors (Cantor et al. 2021; Darling-Hammond et al. 2020; Jamaludin 2024). Understanding how indoor LPP can support cognitive development matters because it is a key component of early childhood education, where indoor play is a dominant part of daily routines. It also matters for parents who seek ways to stimulate their children’s development at home, as loose parts provide flexible, open-ended opportunities for exploration. Given the growing emphasis on play-based learning, research on indoor LPP is essential to inform educators and parents about creating environments that foster meaningful cognitive engagement.

### Current Study

We systematically reviewed the literature reporting on the relationship between young children’s indoor LPP and cognitive development. This systematic review lends insight into whether LPP may be valuable for cognitive development and could be a powerful tool for parents, educators, and policymakers to enhance their children’s early learning environments (Casey and Robertson 2019; Foster 2002). Understanding nuanced ways LPP can support cognitive development can inform more evidence-based educational practices for educators and parents. In addition, this review identifies research priorities for future studies on indoor LPP and cognitive development. Given the limited empirical evidence directly linking indoor LPP with cognitive development in young children, we sought to critically evaluate the quality of the available studies with materials and toys similar to loose parts and identify potential biases or methodological limitations that could impact the interpretation of results. As a result, this addressed two research questions:

1. How has indoor LPP been studied in relation to cognitive development (e.g., study designs and outcome measures)?
2. What is the relationship between indoor LPP and young children’s (0–6) cognitive development and outcomes?

## 2. Methods

The methods for this systematic review were pre-registered on PROSPERO (CRD42023452046).

### 2.1. Search Strategy

Following PRISMA guidelines, a systematic review was conducted in July 2023 and updated in December 2024. A search string, shown in Table 1, was developed and implemented in ERIC, PsycINFO, PsycARTICLES, ScienceDirect, Scopus, CINAHL, Web of Science, JSTOR, ProQuest Theses/Dissertations, Education Research Complete, and Academic Search Complete (see Appendix E for the search strategy adapted for each database).

A variety of databases were searched in the present review to be as comprehensive as possible and to capture articles on LPP published in various disciplines.

### 2.2. Inclusion Criteria

Studies were screened using pre-specified criteria. First, regarding study participants, the article must have reported on a study involving children up to six years old. It was included if a study overlapped with our intended age range (e.g., 5–10). Second, the study must have investigated indoor LPP but could not have studied LPP within arts- or sports-based programs. Indoor LPP was defined as play involving loose parts, including various open-ended materials that could be synthetic, natural, or recycled (Houser et al. 2016). Studies on indoor LPP were only included if they investigated multiple or single materials that were not commercialized. For instance, studies that investigated single materials such as blocks, LEGO, Duplo, Magna-Tiles, Lincoln Logs, Imagination Playground blocks, and Playmobil were not included. We only included branded toys, such as LEGO, DUPLO, Play-Doh, Lite-Brite, etc., when combined with other play materials or toys that could be considered loose parts. Studies of multiple materials could include materials not designated as loose parts. Studies examining play in indoor and outdoor settings were only included if indoor play was disaggregated. Third, play activities could be unstructured or structured (e.g., play scenarios). However, studies were only included if the play observed did not ask children to create art, participate in sports, be involved in competition, or limit play using specific child- or adult-created rules. Using these criteria, we aimed to assess LPP’s free, open-ended and unstructured nature as postulated by LPP Theory (Houser et al. 2016; Nicholson 1971).

Fourth, studies must have examined the relationship between children’s engagement with LPP indoors and children’s cognitive development. The study must have investigated at least one of the following: school/educational achievements, IQ testing, decision-making, problem-solving, executive functioning (e.g., attention, memory, organizing, planning), creativity, divergent thinking, mathematical ability, special ability, Science, Technology, Engineering and Math (STEM) explorations in play, scientific thinking (e.g., gathering evidence, testing hypotheses, drawing conclusions), language, and reading/language comprehension. Assessments of cognitive development must have been based on school/academic reports, standardized testing, or observational assessments conducted by researchers. Studies could report other variables if they focused on the relationship between indoor LPP and cognitive development. For instance, studies looking at home factors impacting children’s developmental outcomes would not be included if there was no separate analysis between indoor LPP and cognitive development. Fifth, studies must have reported on primary research using quantitative (questionnaires, psychological tests, observational sampling, experiments), qualitative, or mixed methods with adequate sampling (e.g., case studies and studies with sample size under 10 were excluded). Sixth, and finally, studies must have been published in English between January 1970 and December 2024 in academic journals or theses/dissertations. The theory of LPP was proposed in the 1970s (Nicholson 1971), and under our inclusion criteria, we aimed to capture any publication within our time frame that examined or referenced loose parts.

### 2.3. Article Selection Process and Inter-Rater Reliability

After initial training, all three authors worked through three stages of inter-rater reliability: (1) title/abstract screening, (2) full-text screening, and (3) data extraction. At the title/abstract stage, all three authors double-coded 10% of the studies. A threshold of 90% reliability was required before coders screened studies individually. At the full-text and data extraction stages, each article was screened by two of the three coders. Disagreements were discussed among all three authors, and decisions were made by reaching a consensus.

### 2.4. Data Extraction and Analysis

The lead author trained co-authors on inclusion and exclusion criteria and data extraction. During training, all three authors piloted 15 test articles (Lombard et al. 2002). Using the included articles, the authors hand-searched reference lists and conducted citation searching. The included articles were narratively synthesized, which involves systematically summarizing the main findings and themes. This approach provided a descriptive overview of the evidence and identified common trends, patterns, and variations. Data were extracted on the study characteristics (e.g., publication year, location of study, participant characteristics), study methods and analytical approach (e.g., sample size, statistical approach), types of play materials used (e.g., natural materials, everyday objects), terms used to describe LPP; domains of and measures used to assess cognitive development, findings regarding the relationship between LPP and cognitive development, and author recommendations for future research.

### 2.5. Risk of Bias and Quality Assessment

Study quality and risk of bias were assessed using the Mixed Methods Appraisal Tool (MMAT) (Hong et al. 2018) as it allows for the assessment of studies using varying methods with one tool. The MMAT starts with two initial questions consistent across all study methods, followed by five specific questions based on the study’s design (i.e., qualitative study, randomized controlled trial (RCT), non-RCT, quantitative descriptive study, and mixed methods). Each item is rated as “Yes”, “No”, or “Cannot Tell”. The lead author and one of the co-authors jointly evaluated the quality of each study.

## 3. Results

### 3.1. Search Results and Study Selection

**Figure 1:**
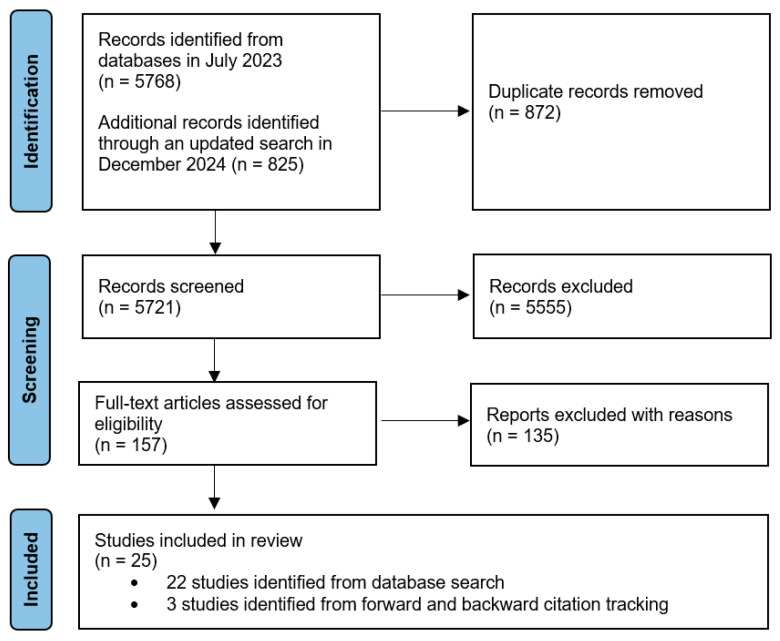
PRISMA flowchart of study screening and selection.

Searches surfaced 6593 articles. Following duplicate removal, 5721 articles remained (see Figure 1). Following title/abstract screening, 157 studies remained for full-text screening. Following a full-text review, a total of 25 studies were included. Reliability between coders exceeded 90% at all stages.

### 3.2. Study Characteristics

Appendix A provides an overview of the key characteristics of the included studies, such as the country where the study was conducted, the age of the children involved, the types of play materials used, and the specific cognitive domains assessed.

### 3.3. Publication Year Distribution

Studies were published between 1976 and 2024. A notable number of studies were published in the 1980s and 1990s, with the most recent studies published in 2024. The early 2000s show a noticeable gap, with only a few studies.

### 3.4. Locations of Studies

Studies were conducted in 14 countries. Most were conducted in the United States (14) and Canada (3). Three studies were conducted across two countries (the United States and the United Arab Emirates). Two studies were conducted in Australia and Thailand, respectively. Single studies were identified from Bangladesh, China, Singapore, Georgia, Denmark, Switzerland, the United Kingdom, Belarus, and South Africa.

### 3.5. Age Distribution of Participants

Most studies concentrated on children between 3 and 6 years old. Four studies included children under two years old, and a few covered a broader age range. None of the studies involved children above six.

### 3.6. Play Materials

Our review included studies investigating a range of play materials, such as blocks, natural elements, sensory materials, and unstructured objects. While most studies incorporated play materials and toys that fit our definition of loose parts, the terms “loose part(s)”, “loose parts play”, or “LPP” were explicitly mentioned in only one of the studies identified (Jaruchainiwat et al. 2024).

### 3.7. Main Findings

How has indoor play with loose parts been studied in relation to cognitive development, focusing on study designs, analysis methods, and the specific cognitive domains explored?

All studies were quantitative. In Appendix B, we outline the methods and data analyses employed. Sample sizes varied considerably across studies. There were a couple of large-scale studies, such as Hamadani et al. (2010), which included 801 participants in Bangladesh and Thepsuthammarat et al. (2012), which included 4116 participants in Thailand. Studies with smaller sample sizes were Morrissey (2014) with 21 participants and Malone et al. (1994) with 22 participants. Sample composition also differed across studies, with some focusing on child dyads (e.g., Morgante 2013) or specific age groups (e.g., Lysyuk 1998).

Observational and cross-sectional studies were most common (e.g., Jaruchainiwat et al. 2024; Lloyd and Howe 2003; Morgante 2013). Some studies had longitudinal designs (e.g., Hamadani et al. 2010; Morrissey 2014; Tomopoulos et al. 2006). While experimental and quasi-experimental studies were less common (O’Connor and Stagnitti 2011; Pepler and Ross 1981; Vieillevoye and Nader-Grosbois 2008), these studies identified relationships using controlled conditions to manipulate variables like play materials or play settings (e.g., indoor and outdoor, Jaruchainiwat et al. 2024).

Regression analyses were commonly used, often incorporating covariates (Hamadani et al. 2010; Luo 2023; Masek et al. 2024; Thepsuthammarat et al. 2012; Vieillevoye and Nader-Grosbois 2008; Wolfgang and Stakenas 1985). For example, Hamadani et al. (2010) employed regression in a longitudinal study to account for factors such as age, household assets, and education, while Luo (2023) utilized structural equation modelling to explore associations in a cross-sectional design, considering variables like gender, age, and family income. Analysis of variance was also widely employed to examine group differences and interactions (Lehman 2014; Liddell and Masilela 1992; McCabe et al. 1996, 1999; Morgante 2013; Pepler and Ross 1981; Saracho 1992). Lehman (2014) used multivariate analysis of covariance (MANCOVA) and multiple analyses of variance (ANOVA) to explore the effects of disability, age, gender, and income in a cross-sectional study, while McCabe et al. (1996) applied repeated-measure multivariate ANOVA (MANOVA) to analyze within-subject variations across multiple variables.

Some studies primarily utilized correlational and frequency analyses to examine relationships and descriptive patterns (Lloyd and Howe 2003; Malone et al. 1994; Morrissey 2014; Rogers 1984; Tizard et al. 1976). For instance, Lloyd and Howe (2003) investigated partial correlations, controlling for variables such as age and sex to explore specific relational dynamics. Similarly, Rogers (1984) employed Pearson correlation to analyze associations across grouped data. Frequency analyses were used in studies like Morrissey (2014) to compare group patterns across sessions, highlighting general trends without testing specific hypotheses. Other studies, such as Lloyd and Howe (2003), Lysyuk (1998), Maker et al. (2023), and Pepler and Ross (1981), utilized frequencies. These studies used frequencies alongside other methods, like correlations and group comparisons, to explore patterns.

### 3.8. Study Quality and Risk of Bias

Overall, the quality of the included studies was high, with only six having a “no” to the question regarding confounders (see Appendix D). All studies had clear research questions and collected data that addressed the research questions. In nine studies, insufficient information was provided to determine whether the sample was representative of the target population.

### 3.9. Cognitive Development and Subdomains of Cognition Studied

We included studies examining how children’s play with specific toys and play materials influences cognitive development or subdomains of cognition such as IQ, creativity, problem-solving, and attention. The outcome measures used ranged from standardized cognitive assessments to observational ratings and analyses of play behaviours (see Appendix C).

Most studies focused on children’s general cognitive development outcomes through standardized assessment tools designed to measure cognitive abilities and intelligence including non-verbal intelligence (Hamadani et al. 2010; Malone et al. 1994; McCabe et al. 1996, 1999; Rogers 1984; Thepsuthammarat et al. 2012; Tomopoulos et al. 2006; Vieillevoye and Nader-Grosbois 2008; Wolfgang and Stakenas 1985). Additionally, several studies used measures that captured multiple cognitive subdomains and skills simultaneously. For instance, McCabe and colleagues (McCabe et al. 1996, 1999) used the McCarthy Scales of Children’s Abilities, which assessed verbal ability, perceptual performance, quantitative skills, memory, and motor development. Malone et al. (1994) included the Battelle Developmental Inventory (BDI) to evaluate cognitive and communication development. Hamadani et al. (2010) used a combination of cognitive and motor measures, including the Mental Development Index (MDI) and the Psychomotor Development Index (PDI).

Language was another significant area of focus (Lehman 2014; Liddell and Masilela 1992; Lloyd and Howe 2003; Luo 2023; Malone et al. 1994; McCabe et al. 1996; O’Connor and Stagnitti 2011; Tizard et al. 1976; Thepsuthammarat et al. 2012; Tomopoulos et al. 2006; Vieillevoye and Nader-Grosbois 2008). Subdomains of language explored included vocabulary (Lehman 2014; Tizard et al. 1976), language comprehension (McCabe et al. 1996; Tizard et al. 1976), total words spoken and syntax (Liddell and Masilela 1992), symbolic naming (Liddell and Masilela 1992), and words and gestures (Hamadani et al. 2010).

Many studies focused on subdomains of cognitive development, exploring the relationship between play and specific skills and capacities. These subdomains included goal setting (Lysyuk 1998; O’Connor and Stagnitti 2011), problem-solving behaviours (Lehman 2014; Maker et al. 2023; Pepler and Ross 1981; Thepsuthammarat et al. 2012), creativity (Jaruchainiwat et al. 2024; Saracho 1992), analytic functioning (Rogers 1984), reasoning (Vieillevoye and Nader-Grosbois 2008), academic skills (i.e., reading and/or math, Lehman 2014; Maker et al. 2023; Masek et al. 2024), attention (Jaruchainiwat et al. 2024), and convergent and divergent thinking (Lloyd and Howe 2003; Pepler and Ross 1981). In addition to exploring cognitive domains or play behaviours within the same study, two studies explored children’s theory of mind, social behaviours, and pretend play competence (Jaggy et al. 2023; Jaruchainiwat et al. 2024; O’Connor and Stagnitti 2011).

We identified many studies focused on children’s play behaviours as the outcome (Jaggy et al. 2023; Lehman 2014; Lloyd and Howe 2003; Malone et al. 1994; Morrissey 2014; Pepler and Ross 1981; Tizard et al. 1976; Trawick-Smith 1990; Saracho 1992). Among these, Trawick-Smith (1990) investigated object transformation during play, and Tizard et al. (1976) examined various play behaviours, types and/or levels of engagement. Uniquely, Jaggy et al. (2023) investigated play behaviours through social pretend play competence and social cognition (i.e., Theory of Mind). Jaggy et al. (2023) found that compared to children in the control condition, children from the material condition had a significantly more positive change in pretend play competence and showed more positive changes in prosocial behaviours reported by educators; however, no differences between the material condition and control group were found for changes in social pretend play competence measured by the Tools of the Play Scale. Although our review does not focus on social development, developing play behaviour competence with various play situations was critical to include, as pretend play behaviours can contribute to cognitive advancement indirectly (Jaggy et al. 2023).

*What is the relationship between young children’s indoor play with loose parts and cognitive development?*

Many of the studies found significant positive associations between play materials and general cognitive development (Lloyd and Howe 2003; Pepler and Ross 1981; Thepsuthammarat et al. 2012; Tomopoulos et al. 2006; Vieillevoye and Nader-Grosbois 2008; Wolfgang and Stakenas 1985). To provide a few examples, Thepsuthammarat et al. (2012) explored the effect of play materials on cognitive outcomes by including 12 different categories of play materials and toys, some of which could be considered loose parts (e.g., home utensils, sound-making toys, junk materials, natural materials, creative materials, self-invented toys, stacking toys). Through a regression analysis, they linked natural and creative materials with improved scores on the Capute Scale, which evaluated problem-solving and language skills. Pepler and Ross (1981) demonstrated that divergent play materials fostered originality and fluency in problem-solving, while convergent materials promoted strategic and task-focused problem-solving, leading to improved accuracy and efficiency. Wolfgang and Stakenas (1985) highlighted that different categories of play materials and toys (e.g., structured constructional toys and macro-symbolic play materials) were associated with distinct cognitive outcome patterns through a regression analysis, including verbal and quantitative development. They emphasized that structured constructional play with materials that maintain their form and shape, such as conventional blocks or LEGO blocks, appears to positively influence verbal, perceptual performance, quantitative, and memory development. Fluid constructional play materials with fluid quality, such as paints or clay that can produce representational products, appear to mainly contribute to perceptual performance. Macro-symbolic play, with child-sized equipment and props used for socio-dramatic play, influences perceptual performance, as well as quantitative and memory development. Thus, different categories of play materials were linked to specific cognitive benefits. Only one standardized measure was shared among a few studies: the McCarthy Scales of Children’s Abilities assessing general cognitive abilities (McCabe et al. 1996, 1999; Wolfgang and Stakenas 1985). Among these studies, while McCabe et al. (1999) and Wolfgang and Stakenas (1985) linked play materials to general cognitive outcomes, McCabe et al. (1996) did not find a relationship.

Some studies explored the relationship between play materials and language development, focusing on specific subdomains such as receptive and expressive language (Tomopoulos et al. 2006), vocabulary, and language comprehension (Lehman 2014). For instance, Tomopoulos et al. (2006) found that fine-motor and symbolic toys significantly predict better language outcomes. Lehman (2014) identified that building toys like blocks were linked to average receptive vocabulary skills in children with developmental delays. In contrast, in the same study, alphabet and language materials supported similar outcomes for typically developing children. Tizard et al. (1976) explored many aspects of play, including play materials; however, they did not correlate with comprehension and expression scores on standardized language assessments. The Peabody Picture Vocabulary Test, measuring vocabulary, was the only common language measure across some studies (Lehman 2014; Lloyd and Howe 2003; Luo 2023; McCabe et al. 1996, 1999). Four of the five studies that explored play materials and their relation to vocabulary found significant relationships, and one did not (Luo 2023).

Several studies explored associations between play materials and toys on various subdomains of cognitive skills (e.g., Jaruchainiwat et al. 2024; Masek et al. 2024). To highlight a few examples, Lloyd and Howe (2003) linked solitary-active play with open-ended materials to improved divergent thinking. Wolfgang and Stakenas (1985) found that constructional and symbolic play materials contributed to analytic functioning, memory, and perceptual development. One study that distinctively explored children’s self-regulation found that pretend play involving symbolic behaviours significantly correlated with better self-regulation in children (Vieillevoye and Nader-Grosbois 2008). The only study that focused on LPP and children’s creativity and attention was conducted by Jaruchainiwat et al. (2024), who found that children’s creativity scores improved between pre- and post-tests. However, these authors defined creativity as exploration, participation, enjoyment, and persistence. Due to the unique goals and measures used and the outcomes discussed, most studies had no common measurement framework. Table 2 provides a summary of the specific cognitive subdomains examined across the included studies. It also indicates the level of agreement among findings when multiple studies investigated the same cognitive subdomain, highlighting areas of consistency or divergence in the research. A summary of all findings is provided in Appendix C.

While many studies found a relationship between children’s use of indoor LPP materials and cognitive outcomes, several studies did not. One study (Liddell and Masilela 1992) reported that fewer words were used when using miscellaneous and school readiness materials (e.g., a large dice, a number, shape and colour sorter), and other toys and materials (i.e., drawings/posters). Five studies reported no significant relationships between play materials and cognitive outcomes (Hamadani et al. 2010; McCabe et al. 1996; Tizard et al. 1976). Hamadani et al. (2010) found that play materials were no longer significant predictors of cognitive or language outcomes when nutritional status and child age were controlled. Similarly, McCabe et al. (1996) also observed no significant effects of play materials on cognitive abilities, language use, or the diversity and complexity of children’s play behaviours. Finally, Jaruchainiwat et al. (2024) found that children’s attention remained unchanged between the pre-test and post-test.

*What covariates or control variables influence the relationship between play materials, play behaviours, and cognitive development?*

Several studies used regression models or partial correlations to analyze the relationship between play materials, behaviours, and cognitive development, at times revealing the critical role of specific covariates such as age, socioeconomic factors, parental education, and home environment (Hamadani et al. 2010; Lehman 2014; Thepsuthammarat et al. 2012; Tomopoulos et al. 2006; Trawick-Smith 1990; Vieillevoye and Nader-Grosbois 2008; Wolfgang and Stakenas 1985). Child-specific factors, such as age and sex/gender, were frequently identified as covariates or control variables (Lloyd and Howe 2003; Wolfgang and Stakenas 1985; Trawick-Smith 1990). In Hamadani et al.’s study (Hamadani et al. 2010), the effect of age was investigated on cognitive outcomes, while sex/gender differences were explored in tasks involving problem-solving (e.g., Pepler and Ross 1981) and memory (e.g., Wolfgang and Stakenas 1985). Only a couple of studies found an age and gender effect on cognitive development in relation to play materials (Trawick-Smith 1990; Wolfgang and Stakenas 1985).

Family and household covariates, including socioeconomic status (SES), parental education, and maternal age, played a crucial role. SES and parental education determined developmental outcomes (Hamadani et al. 2010; Wolfgang and Stakenas 1985). Wolfgang and Stakenas (1985) specifically explored different play materials and their relation to specific cognitive outcomes, finding that SES was predictive of young children’s verbal, quantitative, and memory development scores. Furthermore, health and biological covariates, such as birth weight and disability status, and their influence on cognitive outcomes were explored (Thepsuthammarat et al. 2012). The researchers listed them initially but did not report whether any of these covariates impacted children’s cognitive outcomes or moderated the relationship between play materials and toys and cognitive development in their regression model. Lehman (2014) demonstrated that disability status significantly influenced the relationship between play materials and language outcomes.

## 4. Discussion

### 4.1. Summary of Results

This systematic review identified 25 studies examining the relationship between children’s indoor play with materials and toys that fit the definition of loose parts by Gull et al. (2019) and cognitive development. The majority of these studies reported positive associations between play materials and cognitive outcomes, while five studies did not observe significant relationships. Seven studies did not explicitly analyze the relationship between play materials and cognitive development or its subdomains. Below, we discuss key empirical and methodological gaps identified, highlighting areas for future research to address.

### 4.2. Empirical Gaps in Play Materials and Cognitive Domains Studied

First, many kinds of play materials and toys and cognitive development outcomes are explored across studies. Notably, only one study explicitly used the term “indoor loose parts”. Other studies included play materials and toys ranging from natural and creative materials to symbolic and fine motor toys and structured constructional and macro-symbolic play items. This variability in material options in the studies reflects the diversity of how researchers conceptualize and operationalize the role of play and play materials in cognitive development.

In total, 5 of the 25 studies did not find significant results linking play materials or behaviours to cognitive development (Hamadani et al. 2010; Luo 2023; McCabe et al. 1996; Rogers 1984; Tizard et al. 1976). Also, several studies (Malone et al. 1994; Morrissey 2014; O’Connor and Stagnitti 2011; Saracho 1992; Vieillevoye and Nader-Grosbois 2008) did not focus on the relationship between play materials and toys and cognitive outcomes directly. They examined various play types or behavioural aspects such as pretend play, sequential play, and cognitive style, highlighting the role of play itself as an indicator of cognitive processes. Despite the emphasis that these play types or behaviours were expressions of cognitive processes in their own right, they reflected how play materials shaped children’s behaviours and cognitive processes. We included these studies because they met our inclusion criteria and offered valuable insights into children’s play; however, their analyses or findings did not explicitly articulate the effect of materials. Nonetheless, since materials were embedded in the play context, any observed relationship between play and cognitive development was, by default, could have mediated through the materials used. They challenged clear conclusions regarding play materials and cognitive development outcomes. Taken together, the empirical evidence that shows the link between play materials and cognitive development is limited to a handful of studies within our review.

While many studies focused on children’s general cognitive development, such as IQ, overall mental development, and combined subdomains of cognition (e.g., Hamadani et al. 2010; Thepsuthammarat et al. 2012; Wolfgang and Stakenas 1985), others explored specific domains, including language and academic outcomes. Language development was a significant area of focus, with subdomains such as vocabulary, syntax, and symbolic naming being assessed (e.g., Lehman 2014; Liddell and Masilela 1992; Tizard et al. 1976). Beyond these core areas, several studies focused on subdomains like attention, creativity, problem-solving behaviours, and social cognition, linking play materials to targeted capacities such as cognitive flexibility, inhibition, reasoning, convergent and divergent thinking (e.g., Pepler and Ross 1981; Lloyd and Howe 2003). One subdomain of cognition missing from the research was executive function (EF), which is highly relevant and widely studied in contemporary play research (Doebel and Lillard 2023; Koepp et al. 2022). The relationship between children’s EF skills and play is critical because constructs such as working memory, cognitive flexibility, and inhibitory control are foundational for self-regulation, problem-solving, and learning (Lillard et al. 2013; Whitebread et al. 2012). However, the relationship between EF and play, particularly in the context of LPP, remains underexplored and warrants further investigation. The variability in play materials and the diversity of cognitive domains assessed across studies underscores the potential of play materials and toys to influence a wide range of cognitive skills and the challenges of synthesizing findings to demonstrate consistent impact.

Only two studies focused on children’s mathematical outcomes (e.g., Maker et al. 2023; Masek et al. 2024). Many practitioners and researchers often qualitatively report on the important role of LPP in children’s engagement in STEM behaviours and explorations (Gold et al. 2015; Gull et al. 2024, e.g., building, experimenting, and problem-solving). Gull and colleagues (Gull et al. 2024) explained in detail how loose parts can inspire and encourage children to use their creativity and critical thinking skills in the classroom with science curriculum at any age. Also, a recent teacher action research study by Zeng and Ng (2024) investigated the influence of open-ended questions on five children’s science process skills and the scientific concepts children explore during indoor LPP experiences. Research is needed to determine how loose parts might support STEM learning and explorations across age groups and the specific materials or configurations that encourage scientific and mathematical concepts exploration (Gold et al. 2015). Empirical studies should investigate whether and how indoor loose parts facilitate early engineering skills, spatial reasoning, or scientific inquiry, providing evidence-based guidance for educators and caregivers.

### 4.3. Methodological Gaps

#### 4.3.1. Study Designs

Without targeted and explicit studies on children’s LPP employing longitudinal designs or robust data collection and analysis, drawing conclusions about the role of LPP in children’s cognitive development remains challenging. While longitudinal designs are crucial for understanding the role of play materials in children’s cognitive development, only a few studies have adopted this approach. Drawing conclusions about children’s LPP and its impact is also challenging without robust data collection and analysis that explicitly focuses on LPP. Longitudinal studies on the role of LPP are inherently difficult to conduct due to their extended time frames, significant resource demands, and the complexity of isolating variables over time. However, alternative methodologies, such as highly controlled experimental studies or regression models, can provide valuable insights into the factors influencing children’s engagement with play materials and their cognitive effects. Controlled studies allow for the systematic manipulation of play materials and behaviours while accounting for covariates like age, SES, and parental education, as seen in research by Hamadani et al. (2010). In two different play studies, Hashmi et al. (2021, 2022) randomly provided four alternate boxes of the same materials, demonstrating an innovative approach to exploring how material variation can affect children’s play and cognitive outcomes. By allowing children to engage with multiple material options within the same trial, researchers focusing on LPP could reveal whether certain combinations or types of loose parts promote specific cognitive skills, such as divergent thinking, self-regulation, or executive functioning, more effectively than others. Additionally, this method highlights the importance of flexibility in material choices, ensuring that findings account for how children’s preferences and engagement levels might shift when provided with diverse options, reflecting the real-life application of LPP. Future research should build on this approach and could systematically test material variability within sessions to understand the potential of LPP to support cognitive and developmental outcomes. These approaches can help identify relationships and the contexts in which play materials and toys are most effective.

#### 4.3.2. Covariate Explorations

Many studies explored key covariates or control variables, including age, SES, parental education, and home learning environments. These factors played a critical role in moderating or mediating these relationships, underscoring the importance of considering these factors in future studies. Only a few studies focused on diversity factors such as SES and learning environments (e.g., Luo 2023; Hamadani et al. 2010; Lehman 2014; Wolfgang and Stakenas 1985). These studies underscored the need for research explicitly addressing how covariates can mediate or moderate the impact of play materials and play behaviours on cognitive outcomes. Play is often analyzed through observations and linguistic output (e.g., Liddell and Masilela 1992; Morrissey 2014; Tizard et al. 1976). Given the linguistic, socioeconomic, and cultural diversity in regions such as North America and Europe, studies that examine these variations are crucial for understanding how children from different backgrounds use and potentially benefit from play materials. For instance, cultural norms may influence the types of materials children prefer or how they engage in specific play behaviours (Lin and Li 2020). Similarly, linguistic diversity may shape children’s observed symbolic communication or vocabulary during play. Exploring these dimensions would provide a more nuanced understanding of how play materials support cognitive and social-emotional development across varied contexts, highlighting the importance of culturally responsive approaches in research and practice.

### 4.4. Strengths and Limitations

This review provides a novel contribution to the literature in that it provides the first synthesis of studies that report on the relationship between indoor LPP and children’s cognitive development. LPP is a notable topic of interest to researchers, parents, and educators in early learning environments, so it is important to compile what is known about the value of these play materials. While this review provides valuable information, it has several limitations. First, a few studies directly investigated the relationship between cognitive development, play materials and toys that meet the criteria of loose parts. As a result, the lack of data inhibits firm conclusions about the findings and the relationship between these play materials and children’s development. Second, it is difficult to directly compare study findings due to their methodological heterogeneity (e.g., covariates). Additionally, study heterogeneity restricted the ability to meta-analyze the results.

We selected studies with play materials and toys that met our definition of LPP. However, some studies explored play behaviours, play types, and their impact on cognitive development with minimal focus on materials (Lloyd and Howe 2003; Malone et al. 1994; Morrissey 2014; Pepler and Ross 1981; Tizard et al. 1976; Vieillevoye and Nader-Grosbois 2008). Play behaviours are considered the leading source for development; they may serve as indicators of cognitive outcomes because they reflect children’s ability to articulate their ideas, problem-solve, think creatively, and engage in symbolic thought (Bodrova and Leong 2007; Vygotsky 1967; Whitebread et al. 2012; Yogman et al. 2018). Examining play behaviours to draw conclusions about the relationship between play materials and cognitive outcomes was not always straightforward or reliable. The variability in findings across studies highlights the complexity of isolating the impact of play materials from behaviours on cognitive development. Despite these limitations, play behaviours remain a central focus in some studies, offering insights into children’s cognitive processes.

## 5. Conclusions

This systematic review highlights the complex relationships between play materials, play behaviours, cognitive development, and outcomes, emphasizing the need for further research to address existing gaps. The goal was to explore the relationship between indoor LPP and cognitive development outcomes in young children (ages 0–6). It also examined how indoor LPP has been studied in relation to cognitive development, focusing on study designs and outcome measures. While many studies found a positive, significant relationship between LPP and cognitive outcomes, we identified mixed findings and gaps in the existing literature. Future research should prioritize longitudinal and experimental designs that account for key covariates while incorporating qualitative methods to capture the nuanced ways children interact with loose parts and other toys during play. Greater attention to underexplored areas, such as the role of executive function in children’s play with loose parts and the impact of cultural and linguistic diversity, is essential. Given the variability of loose parts, examining different versions of loose parts play kits may help determine whether these materials consistently support quality play experiences that contribute to cognitive development. Future studies can provide more comprehensive and actionable insights regarding how these materials impact children of different ages and diverse backgrounds by addressing these gaps, ultimately supporting educators, parents, and policymakers to create enriched play environments that support children’s diverse developmental needs. The review identified many underexplored cognitive subdomains and revealed methodological limitations regarding indoor LPP in the current literature. We propose a more integrated research agenda incorporating experimental and longitudinal designs to produce nuanced and reliable evidence explicitly focusing on how loose parts can impact cognitive development in early learning environments and at home before age six. In doing so, this review challenges existing assumptions about how material affordances in early childhood can support cognitive development across diverse contexts.

## Figures and Tables

**Table 1:** Search strategy.

Line 1	(child* OR kid* OR youth* OR minor* OR juvenile* OR early child* OR toddler* OR elementary OR preschool* OR pre-school* OR kindergarten* OR infant* OR bab* OR young)
AND	
Line 2	(“loose part*” OR “play material*” OR “recycle* material*” OR “natural material*” OR “scrap material*”)
AND	
Line 3	(creativ* OR explor* OR cogniti* OR intelligen* OR learn* OR “executive function*” OR knowledge OR skill* OR flexibilit* OR abilit* OR capacit* OR develop* OR achievement* OR outcome* OR problem solv* OR advancement* OR memory OR think* OR attention)

**Table 2:** Cognitive subdomains measured and agreement between study findings.

Studies	Cognitive Subdomains	Agreement Between Findings
Lehman (2014) Pepler and Ross (1981) Thepsuthammarat et al. (2012)	Problem-solving behaviours	Both found a positive relationship between play materials and toys and cognitive subdomains
Jaruchainiwat et al. (2024) Saracho (1992)	Creativity	Both found a positive relationship between play materials and toys and cognitive subdomains
Lehman (2014) Maker et al. (2023) Masek et al. (2024)	Academic skills (reading/math)	All found a positive relationship between play materials and toys and cognitive subdomains
Lloyd and Howe (2003) Pepler and Ross (1981)	Convergent and divergent thinking	Both found a positive relationship between play materials and toys and cognitive subdomains

## Data Availability

No new data were created or analyzed in this study.

## Appendix A. Study Characteristics

**Author**	**Location**	**Child Age**	**Play Materials**
Hamadani et al. (2010)	Bangladesh	Under 2	Toys for playing music, drawing, writing, constructing, stacking, building, moving (e.g., balls, bats), learning shapes and colours, and pretending (e.g., dolls, tea sets). These objects were homemade and found in homes, outside, and toy stores.
Jaggy et al. (2023)	Switzerland	2 to 5 years old	The material condition—a standardized set of firefighter-themed role-play materials, including structured items like helmets and unstructured items like wooden blocks and silk scarfs. New materials were introduced in each session, such as crowns and medical kits, except for the last. The play tutoring condition—same materials, but adults tutored social pretend play. The control group engaged in free play and handicraft activities.
Jaruchainiwat et al. (2024)	Thailand	3 to 5 years old	A photograph of the indoor loose parts kit showed a variety of materials, including pompoms, small pipes, small wooden cubes, yarn balls, stones, etc. The authors did not provide descriptions of the full range of materials.
Lehman (2014)	United States	3 to 5 years old	Blocks, LEGOs, sand, water, commercial, educational toys (e.g., Lite-Brite^©^, puzzles, sorting cups, bead stringing), toy vehicles and work machines (e.g., cars, trains, trucks, backhoe loaders), dress-up items, playhouse, toy kitchen, dishes, and plastic food.
Liddell and Masilela (1992)	South Africa	2 to 6 years old	The study investigated 16 different play materials grouped into six functional categories: School-readiness materials included a number sorter, a shape and colour sorter, and large wooden number dice. Drawing/Posters consisted of large A3 scribbler pads with wax crayons and two child-height conversation posters depicting familiar scenes for black South African children, such as daycare and farm life. Puzzles comprised seven wooden puzzles, sampled twice each, showing familiar scenes like vegetables, a child’s face, and a cow in a field. Construction Blocks included four types of commercially available blocks—Duplo (3), Alex School System (3), Kwikslot (plastic rods that are inserted into one another) (4), and Buksy wooden floor blocks (4). Fantasy Toys featured two dolls (5), four toy cars (5), and a set of small-town replicas with houses, trees, schools, churches, trucks, and people (4). Miscellany included two wheelbarrows (7) and two large multi-coloured balls (7).
Lloyd and Howe (2003)	Canada	4 to 5 years old	Various play materials: open- and closed-ended
Luo (2023)	China	3 to 6 years old	Outdoor physical materials for outdoor environment activities. Various indoor game activity areas and activity materials. The general social-psychological environment between teachers and children communicating and interacting. Curriculum implementation of plan execution, life activities, learning centre activities, and teaching activities. They used colourful pictures and small pictures for language development.
Lysyuk (1998)	Belarus	2 to 4 years old	Small dolls, six wooden blocks of different shapes, clay, a box with coloured pencils, and paper.
Maker et al. (2023)	United States and United Arab Emirates	4 to 6 years old	Variety of materials including instruments (keyboard, ukulele), balls, cones, materials of hats, masks, glasses, various clothing, figures of people and animals, miniature furniture, and blocks of various shapes and colours.
Malone et al. (1994)		2- to 6-year-olds	Three sets of toys for children’s independent play: a mixed set with various play materials (blocks, dolls, animals, trucks, tools, cloth, puzzle), a set focused on doll play (dolls, action figures, nesting cups, mirror, brush, comb, cloth, basket), and a set centred on vehicle play (plane, puzzle people, wagon, blocks, bulldozer, basket). Classroom play: a wide variety of toys (e.g., doll play materials, housekeeping materials, assorted toy vehicles, blocks, puzzles, books, manipulative toys).
Masek et al. (2024)	United States	2 to 3 years old	Four material sets were used: A picture book; a shape sorter, including 12 shapes; a magnet board, including 25 magnetic shapes; and a grocery shopping set, including a cash register, play money and play food items.
McCabe et al. (1996)	United States	5 years old	Study a: For each play activity category, three familiar activities were selected. Functional activities included Styrofoam packing pellets, goop (cornstarch and water), and play dough with tools. Constructive activities involved Lego Duplo blocks, magic markers with paper, and collage-making materials. Dramatic activities featured dress-up clothes with a toy cash register, Fisher-Price playhouses with figures and cars, and toy trains with tracks. Study b: Functional activities included a water table with cups, scoops, goop, and play dough. Dramatic activities featured dress-up clothes with a toy cash register, Fisher-Price playhouses with figures and cars, and toy cars and trucks with a town-themed play mat.
McCabe et al. (1999)	United States	5 years old	Functional play included Styrofoam packing pellets in a cardboard box, “goop” made from cornstarch and water, and Play-Doh with tools. The constructive play involved LEGO DUPLO building blocks, markers, stencils, paper, and collage-making materials with glue. Dramatic play featured dress-up clothes with a toy cash register, Fisher-Price playhouses with people and cars, and Brio trains with train tracks.
Morgante (2013)	United States	3 to 4 years old	Children played at a sensory table filled with either rocks, sand, soil, or water (equal amounts). They were provided with one of two sets of materials stored in clear plastic boxes. Objects in each box were perceptually similar and matched for function (e.g., digging, pouring, containment); they varied in their realism. The minimally structured set included items that loosely represented realistic objects and had multiple uses. Examples from this set included animal and sea animal cookie cutters, plastic tubes, small buckets, wooden dowels, plastic soap dishes, spoons of assorted sizes, doll clothespin-painted people, and wooden block-shaped vehicles. The highly structured set featured more realistic objects, each with a specific function. Examples from this set included animal and sea animal figures, plastic flowerpots, silk flowers, small boats, insect figures, cake pans, Disney’s Little Einstein figurines, garden shovels, and fruit-shaped sponges.
Morrissey (2014)	Australia	Under 2	Various play materials: Level 1: Simple items like a teddy bear, plastic cups and utensils, a small basket, a baby hairbrush, and a tablecloth. Level 2: Expanded to additionally include wooden blocks, material squares, a metal teapot, a doll’s pillow, a plastic truck, and small animal figures (e.g., cat, dog, cow). Level 3: Included similar items as Level 2, but added a doll, assorted wooden blocks, a plastic plate, and a bath duck, offering a more complex and varied set of materials for play.
O’Connor and Stagnitti (2011)	Australia	5 to 8 years old	Four play stations with materials for doll play, transportation, construction, and a home corner
Pepler and Ross (1981)	Canada	3 to 4 years old	Study a: Five distinct sets: animals, vehicles, regular shapes, random shapes, and squares. Additionally, one set consisted of nine different-coloured pieces that fit into a white form board. Study b: Five distinct sets: animals, vehicles, regular shapes, random shapes, and squares. Additionally, one set consisted of nine different-coloured pieces that fit into a white form board.
Rogers (1984)	United States	3 to 6 years old	Play materials and their accessories were contained in 23 different centres: block centre, easel centre, play dough centre, art centre, dress-up centre, water/sand/salt centre, loft centre, building centre, chalkboard centre, puzzle centre, book centre, music centre, toy centre, games centre, workbench centre, puppet centre, science/nature centre, math centre, push-pull centre, perceptual-motor centre, self-propulsion centre, doll centre, and home centre.
Saracho (1992)	United States	3 to 5 years old	Various play materials: small unit blocks, large hollow blocks, block accessories, tricycles, and small pieces of equipment such as puzzles, rods, or peg sets.
Thepsuthammarat et al. (2012)	Thailand	Under 2	Various play materials: push/pull toys, home utensils, sound-making toys, junk materials, dolls and other soft toys, natural materials, storybooks, creative materials, writing materials, self-invented toys, stacking toys, and musical cassettes.
Tizard et al. (1976)	The United Kingdom	3 to 4 years old	Fixed exercise equipment, wheeled vehicles, ladders, large blocks, small construction toys, formboards, paints, clay, sand, dolls, and miniature cars with garages and trains.
Tomopoulos et al. (2006)	United States and United Arab Emirates	Under 2	Rattle, toys on a ring, soft squeeze toy, mirror, soft person or clown, sock rattle, black-and-white pattern items, activity and manipulative toys like pop-up toys, toy instruments, stacking toys, snap beads or links, blocks, push-and-spin toys, and shape sorters. For imaginative play, options include stuffed animals, bath rubber ducks, word-recognition toys, toy telephones, dolls, small cars or trucks, toy radios, and bath boats.
Trawick-Smith (1990)	United States	3 to 6 years old	Toy telephone, wheel of a car, cardboard box, doll, cups.
Vieillevoye and Nader-Grosbois (2008)	United States	2 to 5 years old for typically developing group; 6 to 13 years old developmentally challenged group	Four scenarios with four types of materials: tea party, doctor, transportation, and symbolic creativity.
Wolfgang and Stakenas (1985)	United States	3 to 6 years old	Fluid construction materials (e.g., paints or clay) are malleable, allowing children to create representational products. Structured construction materials (blocks or puzzles) maintain their shape, enabling the creation of representational products. Micro-symbolic materials are small, hand-held toys representing real objects (miniature soldiers, dolls or cars). In contrast, macro-symbolic materials consist of child-sized equipment and props used in socio-dramatic play. Physical materials include objects or equipment (e.g., balls or climbing frames) primarily designed for sensorimotor or physical activities.

## Appendix B. Summary of Methods and Data Analyses

**Citation**	**Study Type**	**Sample Size**	**Data Analysis or Analyses**
Hamadani et al. (2010)	Longitudinal	801	Correlations, frequency distributions, regression (covariates: age, household assets, education)
Jaggy et al. (2023)	Randomized, experimental, pre-test–post-test design	211	Descriptives, correlations, Latent Neighbour Change Model
Jaruchainiwat et al. (2024)	Pre-test–post-test quasi-experimental design	50	T-tests
Lehman (2014)	Cross-sectional	148	MANCOVA, multiple ANOVA, regressions (covariates: disability, age, gender, income)
Liddell and Masilela (1992)	Cross-sectional, observational	Not reported	ANOVA
Lloyd and Howe (2003)	Cross-sectional	72	Frequencies, partial correlations, T-tests (partial corrections controlling for age, sex)
Luo (2023)	Cross-sectional, observational	1642	Correlation and regression (structural equation modelling) (covariates: gender, age, family income)
Lysyuk (1998)	Cross-sectional (grouped by age)	166	Chi-Square, frequencies
Maker et al. (2023)	Cross-sectional, observational	917	Percentages
Malone et al. (1994)	Cross-sectional, randomized	22	Correlational analysis
Masek et al. (2024)	Cross-sectional, within-subjects	42	Logistic regression (covariates: parent age, parent education, toddler age, toddler gender)
McCabe et al. (1996)	Study a and b: Cross-sectional within subjects	Study a: 24 Study b: 24	Study a and a: Multivariate repeated measures ANOVA (MANOVA)
McCabe et al. (1999)	Randomized, quasi-controlled	24	MANOVA
Morgante (2013)	Cross-sectional (dyads by gender)	36	Repeated measures ANOVA
Morrissey (2014)	Non-randomized, longitudinal	21	Frequencies, group comparison across sessions
O’Connor and Stagnitti (2011)	Quasi-experimental design	35	Non-parametric tests, ANCOVA (covariates: baseline age)
Pepler and Ross (1981)	Study a and b: Cross-sectional, experimental	Study a: 64 Study b: 72	Study a and b: Frequencies, ANOVA (covariates: age, sex)
Rogers (1984)	Cross-sectional, grouped by age	49	Pearson Correlation Coefficients
Saracho (1992)	Cross-sectional (over three months)	300	MANOVA (covariates: cognitive style, sex, age)
Thepsuthammarat et al. (2012)	Cross-sectional	4116	Multiple linear regression (covariates: parent factors were age, education, marital status, and income; child factors were sex, weight, height, gestational age, birth weight, breastfed, hospital admission, mother’s attachment, family size, number of siblings, iodine consumption, and life events)
Tizard et al. (1976)	Cross-sectional	109	Correlational, percentages
Tomopoulos et al. (2006)	Longitudinal cohort study	73	Pearson correlations, multiple linear regressions
Trawick-Smith (1990)	Cross-sectional, observational	32	Regression (covariates: sex, age)
Vieillevoye and Nader-Grosbois (2008)	Quasi-controlled experiment	80	Correlations, cluster analysis, multiple regressions (covariate: mental age)
Wolfgang and Stakenas (1985)	Cross-sectional	30	Regression (covariates: age, SES, sex)

## Appendix C. Summary of Cognitive Development and Subdomains Studied, Measures Used, and Findings

**Citation**	**Cognitive Development and Subdomains Studied and Measures Used**	**Findings**	**Summary of the Relationship Between Loose Parts and Cognitive Development**
Hamadani et al. (2010)	Language—MacArthur Communicative Development Inventory (Words and Gestures) Cognitive and Motor—Family Care Indicator (FCI), Mental Development Index (MDI), and Psychomotor Development Index (PDI)	Most regressions found a significant relationship between play materials and cognitive outcomes. The relationship was no longer significant when children’s nutritional status was controlled. Most regressions found a significant relationship between play activities and cognitive outcomes. The relationship was no longer significant when the analyses controlled for child age. The models explained between 16% (psychomotor) and 31% (language comprehension) of the variance in outcomes.	Play materials: no, when nutritional status was considered as a control variable, the predicting power of play materials was no longer significant Play behaviours: no, when age was considered as a control variable, the predicting power of play behaviours was no longer significant.
Jaggy et al. (2023)	The Tools of the Play Scale (ToPS) evaluated children’s ability to substitute objects, actions, speech, and emotions during pretend play. The Playgroup Educator-Reported Social Pretend Play Competence (RPPC) Questionnaire rated children’s pretend play frequency, social involvement, and quality. Educator reports on social behaviour assess children’s empathy, social behaviour, and peer relationship quality. The Extended Theory of Mind Scale (EToM) evaluated children’s understanding of others’ beliefs and desires. The social-emotional competence subtest from the Intelligence and Developmental Scales (IDS-P) evaluated recognition of emotions and understanding of social situations.	Children in the material condition exhibited more positive changes in pretend play competence (reported by playgroup educators). No differences were found for changes in social pretend play competence measured by the ToPS. No differences between the play tutoring condition and material condition were found for changes in social pretend play competence, emotional understanding and ToM, cooperation, sociability, leadership, and setting limits. No differences between material condition and control group were found for changes in social pretend play competence measured by the ToPS, social-cognitive and emotional skills, social behaviour, and positive peer relationships.	Play materials: yes Play behaviours: not explore
Jaruchainiwat et al. (2024)	Creative thinking behaviour was measured using a frequency record of three categories of behaviour: exploration, participation and enjoyment, and persistence (10 items). Attention span was measured using a frequency record of attention.	Post-test scores measuring creative thinking behaviours were significantly greater than pre-test scores. All three categories of creative thinking behaviour were significantly greater in the post-test recordings.	Play materials: yes Play behaviours: not explored
Lehman (2014)	The Woodcock-Johnson Third Edition (WJ-III) measured literacy through the Letter-Word Identification subtest and math achievement (Applied Problems and Quantitative Concepts subtests). The Peabody Picture Vocabulary Test (PPVT) measured receptive vocabulary. Teacher Questionnaires identified the most frequent activities chosen by the child. Parent Questionnaire gathered information on children’s gender, ethnicity, age, and disability type.	Among children with developmental delay, choosing toys like blocks, LEGOs, or K’NEX during free play in preschool is related to average receptive vocabulary skills. For children without disabilities, choosing alphabet and language materials during free play relates to average receptive vocabulary skills in kindergarten. This relationship could not be compared with children with developmental delays, who prefer building toys. Free-play activity choice in preschool is unrelated to student achievement for five-year-old children. Among four-year-old children with developmental delay, choosing to build toys during free play is related to average applied problem-solving skills in first grade. For four-year-olds without disabilities, choosing alphabet and language materials during free play is related to average receptive vocabulary skills in kindergarten. Free-play activity choice is unrelated to five-year-old children’s academic competence, regardless of developmental delays.	Play materials: yes Play behaviours: no, not significant
Liddell and Masilela (1992)	Total speech was calculated by adding the total number of words spoken by adults and children in a ten-minute transcript. Vocabulary was measured as the total number of words spoken in a ten-minute transcript, scored separately for parents and children. Syntax was coded based on grammatical structure, identifying commands, informs, and questions. School-related concepts were coded by identifying utterances that referred to numbers, colours, comparative estimates, or shapes linked to early scholastic achievement.	Children spoke the fewest words when using miscellaneous and school-readiness materials. Other materials led to more speech. Miscellaneous objects were associated with a limited vocabulary compared to a greater vocabulary for other materials. Children most frequently exchanged information when using drawings/posters and least when using miscellaneous objects. School-related concepts were almost exclusively referred to when using school-readiness materials. Children engaged in symbolic naming most often with drawings/posters and least with miscellaneous objects and school-readiness materials.	Play materials: yes Play behaviours: not explored
Lloyd and Howe (2003)	Play: Observed with the Play Observation Scale, tracking how children played and used materials Convergent Thinking: Picture Completion subtest (WISC-R/WPPSI-R) Peabody Picture Vocabulary Test (PPVT-R) Divergent Thinking: Thinking Creatively in Action and Movement Test (TCAM)	Open-ended materials were linked to both intended and non-intended uses, while closed-ended materials were exclusively associated with intended uses, controlling gender and material type. Closed-ended materials and their intended use were positively correlated with PPVT-R. Solitary-active play with the intended use of materials was positively associated with the total TCAM score, fluency subtest, and originality subtest. Solitary-active play with closed-ended materials was positively correlated with the originality subtest. Partial correlations (controlling for gender) indicated that solitary-passive play was significantly and positively associated with open- and closed-ended materials and their intended use. Reticent behaviour (hesitation to engage or reveal thoughts) was negatively related to convergent and divergent thinking measures, with a stronger relationship observed for divergent measures.	Play materials: yes Play behaviours: yes
Luo (2023)	The Path towards Excellence–Chinese Kindergarten Education Quality Rating Standards (PTE-CKEQRS) measured management guidance, environmental support, curriculum promotion, guarantee of teachers’ qualification, and home-kindergarten-community cooperation. Each had several sub-projects with detailed evaluation indicators using the seven-point Likert scale. The Peabody Picture Vocabulary Test (PPVT) measured receptive vocabulary.	Early achievements in language were weakly to moderately correlated with learning centre planning/materials. Additionally, learning centre planning/material was moderately to highly correlated with psychological atmosphere and curriculum implementation quality. Correlations with all four variables and with early achievement in language were positive. Learning centre planning/materials and outdoor venues/facilities did not directly predict children’s early achievement in language.	Play materials: no Play behaviours: not explored
Lysyuk (1998)	Cognitive (Goal setting) no specific measure	Younger children (groups I and II) predominantly engage in activities characterized by a “one toy: one goal” relationship. Significant changes were observed as children aged, with older children (groups III and IV) increasingly demonstrating more complex relationships, such as “different toys: toy-specific goals” and “different toys: one identical goal.”	Play materials: yes Play behaviours: not explored
Maker et al. (2023)	Problem-solving behaviour observations The study assessed children’s abilities across various domains using specific materials and tasks: Auditory: Children repeated rhythms and tones (closed problems) and created original songs or chants using instruments like a ukulele or keyboard (open problem). Bodily/Somatic: Children balanced on one foot and caught a ball (closed), added movements to a series (semi-open), and created movements to accompany music (open). Emotional/Intrapersonal: Children mimicked emotions (closed), interpreted ambiguous emotions in pictures (semi-open), and used props to describe their feelings (open). Linguistic: Children described toys (closed), explained a picture (semi-open), and made up a story with toys (open). Mathematical: Children counted cubes of different colours (closed), grouped attribute blocks by similarity (semi-open), and created their own groups based on different attributes (open). Mechanical/Technical: Children replicated a gear train (closed), then built their own complex gear train with additional materials (open). Moral/Ethical/Spiritual: Children described and resolved a conflict (semi-open) and depicted qualities of a good person (open). Scientific/Naturalistic: Children identified living things in a desert or ocean (closed), described objects (semi-open), and grouped items by characteristics like function or movement (open). Social/Interpersonal: Children were tasked with building a bridge collaboratively (open), while also being assessed on their social interactions and teamwork. Visual/Spatial: Children replicated a construction (closed), built a car (semi-open), and created a unique construction with provided materials (open).	In the auditory/sound, scientific/naturalistic, and emotional/intrapersonal domains, the percentage of problem-solving behaviours consistently increased each year from age 4 to 6. However, in the mechanical/technical, bodily/somatic, and social/interpersonal domains, some behaviours decreased at age five before increasing again at age 6. Similarly, in the linguistic and visual/spatial domains, most behaviours showed an overall increase from age 4 to 6, with the exception of one behaviour in each domain that decreased at age 6. In the mathematical domain, two behaviours decreased at age five and increased at age 6, while one behaviour decreased consistently at age 6.	Play materials: yes Play behaviours: not explored
Malone et al. (1994)	Categorical and Sequential Play Coding Systems were employed to categorize and analyze the types and complexity of children’s play. Play behaviours were coded. Battelle Developmental Inventory (BDI) was used to measure the children’s cognitive and communication development.	For play behaviours, the independent-play condition showed stronger associations between categorical and sequential play behaviours, with children engaging more in sophisticated play sequences at home than in the classroom. Sequential play measures (e.g., multi-scheme sequences and length of play sequences) were positively associated with cognitive, receptive communicative, and expressive communicative developmental ages in both settings.	Play materials: not explored Play behaviours: yes
Masek et al. (2024)	Math words/phrases per minute were calculated based on each of the three types of math talk: numeracy, spatial, or magnitude. Children’s math comprehension was assessed through three tasks: Point-to-Shape, Point-to-Spatial-Relation, and Point-to-X. In each task, children identified the correct picture for each trial.	Children’s total math talk was positively correlated to children’s age. Children were more likely to use numeracy than other forms of math talk. Children’s math talk was statistically different between materials and was greatest when playing with the grocery shopping set, even when accounting for parent sex and parent dominant language. Numeracy talk was more frequent with the picture book and grocery shopping set than with the shape sorter and magnet board. Spatial talk was more frequent when playing with the magnet board and shape sorter than with the grocery shopping set and picture book. Children were likelier to use magnitudes when playing with the grocery shopping set and the magnet board than with the picture book. They were also more likely to use magnitudes with the magnet board than the shape sorter. Children’s overall math talk showed moderate to large effect sizes on toddlers’ spatial understanding.	Play materials: yes Play behaviours: not explored
McCabe et al. (1996)	Study a and Study b: The McCarthy Scales of Children’s Abilities (McCarthy) assesses general cognitive abilities. The Peabody Picture Vocabulary Test-Revised (PPVT-R) measured receptive vocabulary. The Test for Auditory Comprehension of Language-Revised (TACL-R) includes two sections, Grammatical Morphemes and Elaborated Sentences, used to assess language use.	Study a and Study b: No significant results. The category of play materials did not affect the amount, diversity, or complexity of language used.	Study a and Study b: Play materials: no, not significant Play behaviours: not explored
McCabe et al. (1999)	The McCarthy Scales of Children’s Abilities (McCarthy) assesses general cognitive abilities. The Peabody Picture Vocabulary Test-Revised (PPVT-R) measures receptive vocabulary.	The category of play materials had a significant effect. Functional, constructive, and dramatic play materials each elicited more of their respective types of play and amount of play. Specific toys within each category varied in their effectiveness in engaging children and eliciting expected play types: DUPLOs and markers were effective for constructive play. Dress-up clothes and Fisher-Price houses were effective for dramatic play. The choice of specific toys within each category influenced the type and amount of play observed. The McCarthy GCI and PPVT-R were negatively correlated to the amount of functional play overall and functional play with constructive and dramatic materials. The McCarthy was positively correlated to dramatic play with functional materials whiel the PPVT-R was positively related to overall dramatic and constructive play as well as constructive play with constructive materials. The percentage of dramatic play also accounted for the variance in the number of different words used.	Play materials: yes Play behaviours: yes
Morgante (2013)	The Play Observation Scale was used to record the predominant cognitive play form and social context.	The surface of the sensory table significantly influenced various types of play behaviours. Functional, constructive, and dramatic play all showed main effects based on the surface type, with water encouraging the most functional play and sand and soil promoting the most constructive play. Dramatic play occurred more frequently with water than with rocks. Highly structured objects led to more dramatic play, while minimally structured objects fostered more functional play. Interestingly, highly structured objects, expected to increase social play, instead pulled for more solitary-constructive and solitary-dramatic play. Minimally structured objects promoted greater socialization through parallel and social play and social-constructive play when nested. One significant interaction was found between objects and dyads in parallel-functional play, where heterogeneous dyads engaged in more parallel-functional play with the minimally structured object set than homogeneous male-male dyads. Several significant interactions between surface and object types were observed, particularly in constructive and parallel play. For instance, coupling sand with highly structured objects increased constructive play in heterogeneous dyads, whereas sand paired with minimally structured objects increased parallel play.	Play materials: yes Play behaviours: not explored
Morrissey (2014)	The Pretend Play Observation Scale measured the stages of pretend play in both children and mothers. For children, it assessed the most advanced level of pretend play they showed, from simple actions to complex transformations. For mothers, it focused on their role in modelling and supporting play, evaluating the highest level of pretend play they demonstrated, excluding planning and simple actions. The scale looked at actions and verbal cues to determine the play level.	Children showed a significant increase in the frequency and complexity of pretend play behaviours across three sessions. Play levels advanced from early stages (Stage 1) in Session 1, to higher stages (up to Stage 10) by Session 3, with a noticeable decline in lower-level pretend play and an emergence of more complex play behaviours. Mothers initially engaged in more play behaviours than their children. Still, by Session 3, children’s play frequencies matched or exceeded those of their mothers, reflecting a typical scaffolding pattern where responsibility for play shifts from mother to child. Mothers demonstrated play at more advanced levels than their children, with play ranging from Stages 2 to 10 across all sessions. The gap between mothers’ and children’s play levels narrowed over time, aligning with the scaffolding model. Mothers often modelled play several levels ahead of their children’s abilities rather than just one level ahead.	Play materials: not directly explored Play behaviours: yes
O’Connor and Stagnitti (2011)	The Child-Initiated Pretend Play Assessment (ChIPPA) evaluates a child’s ability to engage in pretend play by initiating and sustaining play scenarios. The Preschool Language Scale, Fourth Edition (PLS-4) was used to assess language development, including receptive and expressive language skills. Goal Attainment Scaling (GAS) was used to assess the achievement of specific goals set for an individual. The Penn Interactive Peer Play Scale (PIPPS) was used to assess children’s interactive play behaviours with peers, focusing on their social competence and the quality of peer interactions.	Significant improvements in children’s ability to engage in pretend play (ChIPPA), language, and goal attainment in the play intervention group. When children’s results were explored with baseline scores and age-controlled, there were no significant improvements for the control or intervention group.	Play materials: not directly explored Play behaviours: no, not significant
Pepler and Ross (1981)	Study a: Play sessions were conducted and coded for the following play behaviours: Form-Related, Colour-Related, Representation-Related, Investigation-Related, Construction-Related, and Symbolic. Divergent problem-solving tasks (Village and Structure-Meaning Task) evaluated fluency and originality in generating structures and labels. Convergent problem-solving tasks (Form-Matching, Colour-Matching, and Representation-Matching) measured the number of correct responses and time taken, focusing on accuracy and efficiency. Study b: Play sessions coded for the same play behaviours as study 1. Divergent problem-solving tasks included the same Structure-Meaning Task from study 1 and the Multiple-Use Task, which assessed the originality and number of verbal and demonstrative responses. Convergent problem-solving tasks included four puzzles designed to be matched based on specific cues (form, representation, colour) while disregarding irrelevant distracting cues.	Study a: Children in the convergent play condition focused primarily on the task. In contrast, those in the divergent play condition produced more responses, which were also more unique and engaged in a wider range of behaviours, including investigation, construction, grouping by properties, and symbolic play. The divergent play group demonstrated the highest originality on the structure-meaning task and contributed to increased fluency in divergent problem-solving tasks. Study b: Children in the divergent play condition engaged in more investigative behaviours, such as exploring rolling properties. In contrast, children in the convergent play group spent most of their time on convergent activities. On divergent problem-solving tasks, the divergent play group produced more unique responses than the convergent and control groups, indicating higher originality in problem-solving. There were no significant effects of age or sex. The convergent play group outperformed the control group on form and representation puzzles, requiring fewer runs to complete them.	Study a: Play materials: yes Play behaviours: yes Study b: Play materials: yes Play behaviours: yes
Rogers (1984)	The Thinking Creatively in Action and Movement (TCAM) was used to measure creative ability. The Preschool Embedded Field Test (PEFT) measures cognitive styles of field dependence-independence and analytic functioning.	The results of the present study revealed that the purported relationships between the TCAM variables of fluency, originality, and imagination and the use of unstructured and structured play materials were insignificant. The study did not find significant gender differences in the relationships between TCAM variables and the use of unstructured and structured play materials.	Play materials: no, not significant Play materials: not explored
Saracho (1992)	Play Rating Scale (PRS) measures children’s play by assessing frequency, creativity in communicating ideas, social interaction levels, and engagement in dramatic play The Preschool Embedded Figures Test (PEFT) measures Field-Dependence/Independence	Children with field-independent (FI) cognitive style engaged more in play than field-dependent (FD) children, highlighting the impact of cognitive style on play behaviours. Significant interactions were also found between age and play behaviours, sex and play behaviours, sex and cognitive style, and among age, sex, and play behaviours. The analysis revealed that FI and FD children differed significantly in all play behaviours except frequency of play.	Play materials: not directly explored Play behaviours: yes
Thepsuthammarat et al. (2012)	The Capute scale consists of a Cognitive Adaptive Test (CAT) and Clinical Linguistic and Auditory Milestone Scales (CLAMS). The CAT is used to evaluate fine motor and problem-solving skills, while the CLAMS determine language skills.	Five types of play materials remained significantly associated with higher Capute scale scores after controlling for other factors: natural materials, creative materials, push/pull toys, sound-making toys, and storybooks. Natural materials had the most significant associations with the Capute scale scores, followed by creative materials and pull and push toys. No mention of covariates and how they affected the regression results.	Play materials: yes Play behaviours: not explored
Tizard et al. (1976)	Observations of children’s play by measuring duration, organization, material use, social participation, play sequence length, and symbolic themes to assess cognitive and social aspects of their play behaviours. Reynell Developmental Language Scale for language comprehension and expression. Minnesota Non-verbal Intelligence Scale for non-verbal cognitive abilities	There were no consistent correlations between aspects of play and verbal and non-verbal standardized test scores.	Play materials: no Play behaviours: no
Tomopoulos et al. (2006)	The Bayley Scales of Infant Development (MDI) assess cognitive development, focusing on attention, memory, problem-solving, and language skills. The Preschool Language Scale-3 (PLS-3) evaluates receptive and expressive language abilities in children from birth to 6 years and 11 months, helping to identify potential language delays or disorders. Additionally, the Caregiver-Child Interaction Rating Scale observes and rates the quality of caregiver-child interactions, emphasizing aspects such as responsiveness, warmth, and stimulation to understand their influence on child development outcomes.	Symbolic and Fine Motor/Adaptive toys were significant predictors of better language outcomes, and the presence of Fine Motor/Adaptive toys was associated with a decreased likelihood of early intervention eligibility at 21 months. Toys at 18 months were significantly associated with maternal language input but not with mutual communication, which mediated the relationship between resources in the home and developmental outcomes. Including parent–child verbal interactions in regression models reduced the degree to which resources (books and toys) were related to developmental outcomes.	Play materials: yes Play behaviours: not explored
Trawick-Smith (1990)	Transformation observations Type I: Using an object in its conventional way but within a make-believe context. For example, using a toy telephone as if making an actual call or using a cardboard box as if it were a box of animal crackers. Type II: Giving an object a completely new make-believe identity by using it in a way different from its intended function. For example, using a plate as a car’s steering wheel or a wooden rod as an oar for an imaginary boat. Type III: Using a body part or gesture to represent an absent object. For instance, forming a hand as if holding a cup and drinking imaginary water.	More object transformations occurred within realistic environments. Neither age nor sex showed a significant relationship with overall transformations. Type I Transformations were more frequent in realistic play settings, regardless of age or sex. Type II Transformations occurred significantly less often in realistic environments. Until age five, realistic materials elicited the greatest number of overall transformations. After age five, the frequency in non-realistic environments surpassed that in realistic settings. An interaction between age, the realism of play objects, and sex was observed for overall transformations. This interaction was primarily due to a strong correlation between age and transformations for girls in non-realistic environments. For boys, the relationship was less pronounced and not significant, suggesting that realistic materials were more effective in eliciting object transformations among boys even after age five.	Play materials: yes Play behaviours: yes
Vieillevoye and Nader-Grosbois (2008)	The Snijders-Oomen Non-Verbal Intelligence Test (SON) assesses cognitive development, focusing on reasoning and performance through six subtests. It scores mental age, intellectual, reasoning, and performance quotients. The Language Evaluation (ELO) evaluates language skills across vocabulary, phonology, comprehension, and linguistic production, using six subtests that measure receptive and expressive language abilities. The Test of Pretend Play (ToPP) measures various forms of pretend play, assessing object substitution, imaginative acts, and the use of objects or agents in pretend scripts.	Individual and dyadic pretend play explained self-regulation in children of both the typically developping and disability groups. Specifically, in both groups, the higher the symbolic behaviour in the creativity context, the higher self-regulation.	Play materials: not directly explored Play behaviours: yes
Wolfgang and Stakenas (1985)	McCarthy Scales of Children’s Abilities: assessed verbal ability, perceptual performance, quantitative development, memory, and motor development. Play Form Scale explored a form of play (i.e., and the toys associated with it in the home environment. The Play Forms Scale includes five subdomains: Fluid Materials, Structured Materials, Microsymbolic, Macrosymbolic, and Physical. Data on all toys in each child’s home (indoors and outdoors) were collected and catalogued.	The study demonstrated a relationship between toys in the home environment and cognitive development. Findings suggest that different toys and play forms relate to different cognitive development patterns. Structured constructional play/materials influenced perceptual performance, verbal, quantitative, and memory development. Fluid constructional play/materials contributed mainly to perceptual performance, while macro-symbolic play/materials influenced perceptual performance and quantitative and memory development. Micro-symbolic play/materials enhanced memory.	Play materials: yes Play behaviours: yes

## Appendix D. Study Risk of Bias and Quality—MMAT Results

**Citation**	**For All Types of Studies**		**Quantitative Randomized**					**Quantitative Non-Randomized**					**Quantitative Descriptive**					**Mixed Methods**				
	**S1**	**S2**	**2.1**	**2.2**	**2.3**	**2.4**	**2.5**	**3.1**	**3.2**	**3.3**	**3.4**	**3.5**	**4.1**	**4.2**	**4.3**	**4.4**	**4.5**	**5.1**	**5.2**	**5.3**	**5.4**	**5.5**
Hamadani et al. (2010)	Yes	Yes						Yes	Yes	Yes	No	Yes	Yes	Yes	Yes	Yes	Yes					
Jaggy et al. (2023)	Yes	Yes	Yes	Yes	Yes	Yes	Yes						Yes	Yes	Yes	Yes	Yes					
Jaruchainiwat et al. (2024)	Yes	Yes						Yes	Yes	Yes	No	Yes										
Lehman (2014)	Yes	Yes						Yes	Yes	Yes	Yes	Yes						Yes	Yes	Yes	Yes	Yes
Liddell and Masilela (1992)	Yes	Yes						Yes	Yes	Yes	No	Yes	Yes	Yes	Yes	Yes	Yes					
Lloyd and Howe (2003)	Yes	Yes						Yes	Yes	Yes	No	Yes	Yes	Yes	Yes	Yes	Yes					
Luo (2023)	Yes	Yes											Yes	Yes	Yes	Yes	Yes					
Lysyuk (1998)	Yes	Yes						Yes	Yes	Yes	No	Yes	Yes	Yes	Yes	Yes	Yes					
Maker et al. (2023)	Yes	Yes						Yes	Yes	Yes	Yes	Yes	Yes	Yes	Yes	Yes	Yes					
Malone et al. (1994)	Yes	Yes						Yes	Yes	Yes	Yes	Yes	Yes	X	Yes	Yes	Yes					
Masek et al. (2024)	Yes												Yes	X	Yes	Yes	Yes					
McCabe et al. (1996)	Yes	Yes						Yes	Yes	Yes	Yes	Yes	Yes	X	Yes	Yes	Yes					
McCabe et al. (1999)	Yes	Yes	X	Yes	Yes	Yes	Yes						Yes	X	Yes	Yes	Yes					
Morgante (2013)	Yes	Yes						Yes	Yes	Yes	Yes	Yes	Yes	Yes	Yes	Yes	Yes					
Morrissey (2014)	Yes	Yes						Yes	Yes	Yes	Yes	Yes	Yes	Yes	Yes	Yes	Yes					
O’Connor and Stagnitti (2011)	Yes	Yes	Yes	Yes	Yes	X	Yes	Yes	Yes	Yes	Yes	Yes	Yes	Yes	Yes	Yes	Yes					
Pepler and Ross (1981)	Yes	Yes	Yes	Yes	Yes	N/A	Yes						Yes	Yes	Yes	Yes	Yes					
Rogers (1984)	Yes	Yes						Yes	Yes	Yes	Yes	Yes	Yes	Yes	Yes	Yes	Yes					
Saracho (1992)	Yes	Yes						Yes	Yes	Yes	Yes	Yes	Yes	Yes	Yes	Yes	Yes					
Thepsuthammarat et al. (2012)	Yes	Yes						Yes	Yes	Yes	Yes	Yes	Yes	Yes	Yes	Yes	Yes					
Tizard et al. (1976)	Yes	Yes						Yes	Yes	Yes	No	Yes	Yes	Yes	Yes	Yes	Yes					
Tomopoulos et al. (2006)	Yes	Yes						Yes	Yes	Yes	Yes	Yes	Yes	Yes	Yes	Yes	Yes					
Trawick-Smith (1990)	Yes	Yes						Yes	Yes	Yes	Yes	Yes	X	X	Yes	Yes	Yes					
Vieillevoye and Nader-Grosbois (2008)	Yes	Yes						Yes	Yes	Yes	Yes	Yes	Yes	Yes	Yes	Yes	Yes					
Wolfgang and Stakenas (1985)	Yes	Yes						Yes	Yes	Yes	Yes	Yes	Yes	X	Yes	Yes	Yes					
Note: Cells denoted by ‘X’ indicate that the presence or absence of the factor could not be determined based on the information provided in the article.																						

## Appendix E. The Search Strategy Adapted for Each Database

**Search Database**	**Search Results**	**Search Platform Used**	**Search Fields and Restrictions**	**Search Words**	**Noted Information/Explanation**
Academic Search Complete	271 hits	EBSCO host	Search Field: -default fields (search authors, abstract, title, keywords, subjects) -Boolean operator Restricted Search: -publication from start of January 1970- end of December 2023 -English only -articles, reports, dissertations, books	(child* OR kid* OR youth* OR minor* OR juvenile* OR toddler* OR elementary OR preschool* OR pre-school* OR kindergarten* OR infant* OR bab* OR young) **AND** (“Loose part*” OR “play material*” OR “recycle* material*” OR “natural material*” OR “scrap material*”) **AND** (creativ* OR explor* OR cogniti* OR intelligen* OR learn* OR “executive function*” OR knowledge OR skill* OR flexibilit* OR abilit* OR capacit* OR develop* OR achievement* OR outcome* OR problem solv* OR advancement* OR memory OR think* OR attention)	-used platform to search across multiple database -advanced search allows to choose fields and restrictions -no word restriction -made the decision to choose default search as choosing all text resulted in over 20,000 hits, and when specified individual search fields it excluded important articles and significantly decreased the amount of hits
APA PsychArticles	23 hits				
APA PsychInfo	326 hits				
ERIC	163 hits				
Education Research Complete	140 hits				
CINAHL	66 hits				
Ejournals	332 hits				
Scopus	1265 hits		Search Field: -Boolean operator Restricted Search: -publication < 1969 -English only -Articles/Reviews	(ALL (child* OR kid* OR youth* OR minor* OR juvenile* OR early AND child* OR toddler* OR elementary OR preschool* OR pre-school* OR kindergarten* OR infant* OR bab* OR young) AND ALL (“Loose part*” OR “play material*” OR “recycle* material*” OR “natural material*” OR “scrap material*”) AND ALL (creativ* OR explor* OR cogniti* OR intelligen* OR learn* OR “executive function*” OR knowledge OR skill* OR flexibilit* OR abilit* OR capacit* OR develop* OR achievement* OR outcome* OR problem AND solv* OR advancement OR memory OR think* OR attention)) AND PUBYEAR > 1969 AND (LIMIT-TO (LANGUAGE, “English”)) AND (LIMIT-TO (DOCTYPE, “ar”) OR LIMIT-TO (DOCTYPE, “re”))	
JSTOR	1550 hits	JSTOR database	Search Field: -all fields (abstract, item title, author, caption) -Boolean operator Restricted Search: -Access type (everything) -English only -Journals and Book chapters -1 January 1970 to 31 December 2023	(child*)) AND ((“Loose part*” OR “play material*” OR “recycle* material*” OR “natural material*” OR “scrap material*”))) AND ((creativ* OR explor* OR cogniti* OR intelligen* OR learn* OR “executive function*” OR knowledge OR skill*)	-database had a restriction on the number of keywords that could be searched which prevented us from searching all terms. Terms narrowed down
Science Direct	1081 results		Search Field: -search articles with selected key terms -Boolean operator Restricted search -1970–2023 -Journals, book chapters, encyclopedias	(child OR children) AND (“Loose parts” OR “play material” OR “recycle material” OR “natural material” OR “scrap material) AND (cognitive)	-database had a restriction of the number of keywords that can be searched which prevented us from searching all the terms -terms narrowed down -does not allow truncation of words -does not have an English only specification -checked to make sure 1970–2023 captured articles IN both 1970 and 2023
Web of Science	551 results	Clarivate	Search Field: -search terms in all fields -Boolean operator Restricted Search: -1970–2023 -articles -English only	ALL = ((child* OR kid* OR youth* OR minor* OR juvenile* OR toddler* OR elementary OR preschool* OR pre-school* OR kindergarten* OR infant* OR bab* OR young) AND (“Loose part*” OR “play material*” OR “recycle* material*” OR “natural material*” OR “scrap material*”) AND (creativ* OR explor* OR cogniti* OR intelligen* OR learn* OR “executive function*” OR knowledge OR skill* OR flexibilit* OR abilit* OR capacit* OR develop* OR achievement* OR outcome* OR problem solv* OR advancement* OR memory OR think* OR attention))	-checked to make sure 1970–2023 captured articles IN both 1970 and 2023
